# Supplementary material for: NOS inhibition sensitizes metaplastic breast cancer to PI3K inhibition and taxane therapy via c-JUN repression
Source: Nat Commun. 2024 Dec 30;15:10737. doi: 10.1038/s41467-024-54651-x (PMC11685991; doi:10.1038/s41467-024-54651-x)
Supplement: Supplementary file 1 — Supplementary Information [file 41467_2024_54651_MOESM1_ESM.docx]

**Supplementary Information:**

**Supplementary Table 1. Overview of MpBC Patient Characteristics analyzed from Phase 1B/2 Clinical Trial**

| **Patient ID** | **Responder (R)/**  **Non-Responder (NR)** | **Overall Response** | **Tumor Pathological Features** |
| --- | --- | --- | --- |
| 100-004 | R1 | SD | Squamous Differentiation |
| 100-009 | NR1 | PD | Spindle Cell Neoplasm |
| 100-010 | NR2 | PD | High Grade Spindle Cell Neoplasm |
| 100-013 | R2 | PR | Spindle Cell Neoplasm |
| 100-018 | NR3 | PD | Keratinized MpBC |
| 100-024 | R3 | PR | High Grade Neoplasm with Focal Myxo-Chondroid Features |
| 100-027 | NR4 | PD | Spindle Component, keratinizing Squamous Component, Osteosarcomatous Component |
| 100-028 | NR5 | PD | Metaplastic Carcinoma |
| 100-029 | NR6 | PD | Squamous Differentiation |
| 100-048 | R4 | CR | Metaplastic Carcinoma |

**Supplementary Table 2. Association among Ethnicity, Body Mass Index (BMI), and Pathological Features with Response.**

|  | **Total (n=13)** | **Non-Responder** | **Responder** | **P-value (Fisher’s Exact)** |
| --- | --- | --- | --- | --- |
| **Ethnicity**  Caucasian  Other | 10 (76.92%)  3 (23.08%) | 4 (57.14%)  3 (42.86%) | 6 (100%)  0 (0.00%) | **0.19** |
| **Obesity**  <25 BMI  ≥25 BMI | 3 (23.08%)  10 (76.92%) | 2 (28.57%)  5 (71.43%) | 1 (16.67%)  5 (83.33%) | **1.0** |
| **Pathological Features**  Spindle  Other | 5 (38.46%)  8 (61.5%) | 4 (57.14%)  3 (42.86%) | 1 (16.67%)  5 (83.33%) | **0.27** |

Data are presented as n (%). P-value derived from two-sided Fisher’s Exact Test. Two patients with adverse events were excluded from the analysis.

**Supplementary Table 3. Media used for cell lines**

| **Cell Line** | **Media** |
| --- | --- |
| Hs578T | DMEM |
| SUM159 | DMEM |
| MDA-MB-231 | DMEM |
| MDA-MB-468 | DMEM |
| JIMT1 | DMEM |
| ZR-7530 | RPMI |
| T47D | RPMI |
| HCC1937 | RPMI |
| HCC1954 | RPMI |
| HCC38 | RPMI |
| BT549 | RPMI |
| BT20 | EMEM |
| MCF-7 | EMEM |
| SKBR3 | McCoy’s Medium |
| MCF-10A | DMEM containing 10% FBS, 10 μg/mL insulin (Gibco), 0.5 μg/mL hydrocortisone (Stem Cell), and 10 ng/mL EGF (Sigma). |

No commonly misidentified cell lines were used in the study.

**Supplementary Table 4: qRT-PCR Primers**

| Gene | Sequence (5’-3’) |
| --- | --- |
| *NOS2* | F: CAATGTGGAGAAAGCCCCCT  R: GCATCCAGCTTGACCAGAGA |
| *Xbp1* | F: GCTCAGACTGCCAGAGATCG  R: GCGCTGTCTTAACTCCTGGT |
| *Fos* | F: GGGGCAAGGTGGAACAGTTA  R: AGTTGGTCTGTCTCCGCTTG |
| *Jun* | F: GTGCCGAAAAAGGAAGCTGG  R: CTGCGTTAGCATGAGTTGGC |
| *Creb3* | F: AGAGGGGACCCAGATGACTC  R: AATCTTCCTCCGCACACGTT |
| *Lcn2* | F: GTGAGCACCAACTACAACCAGC  R: GTTCCGAAGTCAGCTCCTTGGT |
| *Tgfb1* | F: TTGACTTCCGCAAGGACCTC  R: CCCGGGTTATGCTGGTTGTA |
| *Gapdh* | F:CGGGGCTCTCCAGAACATCATCC  R:GACGCCTGCTTCACCACCTTCTTG |

**Supplementary Table 5. Antibodies used in immunoblotting**

| Antibody | Vendor | Catalog Number | Concentration |
| --- | --- | --- | --- |
| iNOS | Novus Biologicals | NBP1-33780 | 1:1500 |
| p-Akt (Ser473) | Cell Signaling | 4060 | 1:1000 |
| p-Akt (Thr308) | Cell Signaling | 4056 | 1:500 |
| PTEN | Cell Signaling | 9559 | 1:1000 |
| Akt | Cell Signaling | 4691 | 1:1000 |
| p-S6 | Cell Signaling | 4858P | 1:1000 |
| S6 | Cell Signaling | 2217S | 1:1000 |
| γ-H2AX | Cell Signaling | 9718S | 1:1000 |
| Rad51 | Cell Signaling | 8875 | 1:1000 |
| Chk1 | Cell Signaling | 2360 | 1:1000 |
| Chk2 | Cell Signaling | 6334 | 1:1000 |
| ZO-1 | Cell Signaling | 8193 | 1:1000 |
| E-cadherin | Cell Signaling | 3195 | 1:1000 |
| Vimentin | Cell Signaling | 5741 | 1:1000 |
| Zeb1 | Cell Signaling | 3396 | 1:1000 |
| HSP90 | Cell Signaling | 4877 | 1:3000 |
| TGFβ (latent) | Cell Signaling | 3711 | 1:1000 |
| TGFβ (active form) | Santa Cruz | Sc-130348 | 1:1000 |
| LCN2 | Cell Signaling | D4M8L | 1:1000 |
| JNK | Cell Signaling | 9252 | 1:1000 |
| c-Jun | Cell Signaling | 9165 | 1:1000 |
| p-c-Jun (Ser73) | Cell Signaling | 3270 | 1:1000 |
| p-c-Jun (Ser63) | Cell Signaling | 91952 | 1:1000 |
| GSNOR | Abcam | Ab177932 | 1:5000 |
| Tubulin | Cell Signaling | 5346 | 1:3000 |
| GAPDH | Cell Signaling | 3683 | 1:3000 |
| Anti-Rabbit IgG, HRP-linked | Cell Signaling | 7074 | 1:3000 |

**Supplementary Table 6. Antibodies used in IHC/IF Analysis**

| **Antibody** | **Vendor** | **Catalog Number** | **Concentration** |
| --- | --- | --- | --- |
| iNOS | Novus Biologicals | NBP1-33780 | 1:50 |
| Phospho-Akt Ser473 | Cell Signaling | 4060 | 1:50 |
| PTEN | Cell Signaling | 9559 | 1:50 |
| E-cadherin | Abcam | ab76055 | 1:100 |
| Zeb1 | Cell Signaling | 70512 | 1:100 |
| ALDH1 | Abcam | ab52492 | 1:200 |
| Cleaved Caspase 3 | Cell Signaling | 9661 | 1:50 |

**Supplementary Figure** **1**
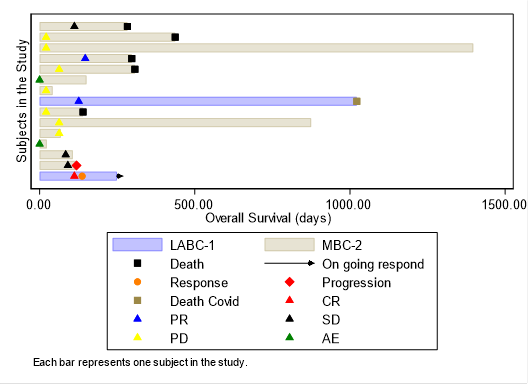


**A**


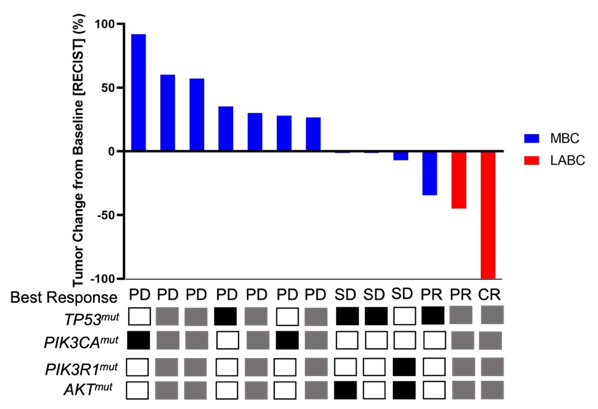


**B**


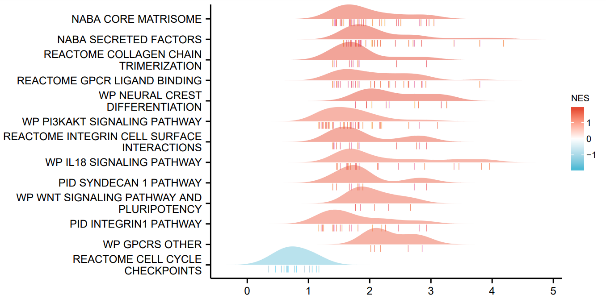

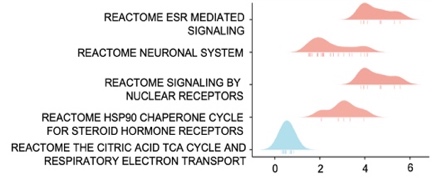


**C**

Downregulated Gene Sets

Metaplastic BC vs Invasive Ductal Carcinoma

**Supplementary Figure 1. (A)** Swimmer’s plot summarizing all the patients in the study. Each bar represents 1 subject in the study. Bar length represents time on study.  **(B)** Waterfall plot from Figure 7A with associated *TP53/PIK3CA/PIK3R1/AKT1* mutation status in 13 patients with available data from post baseline assessment. Blue bars in waterfall plot indicate metastatic breast cancer (MBC) and red bars indicate locally advanced breast cancer (LABC). Black boxes below waterfall plot indicate mutation present, white boxes indicate wild-type/proficient, grey boxes indicate data not available. **(C)** Gene set enrichment analysis of the top represented downregulated hallmark gene sets based on normalized enrichment score (NES) from RNA-sequencing data collected from TCGA in human MpBC tumors (n=14) compared to invasive ductal carcinoma (IDC) tumors (n=814).

**Supplementary Figure** **2**


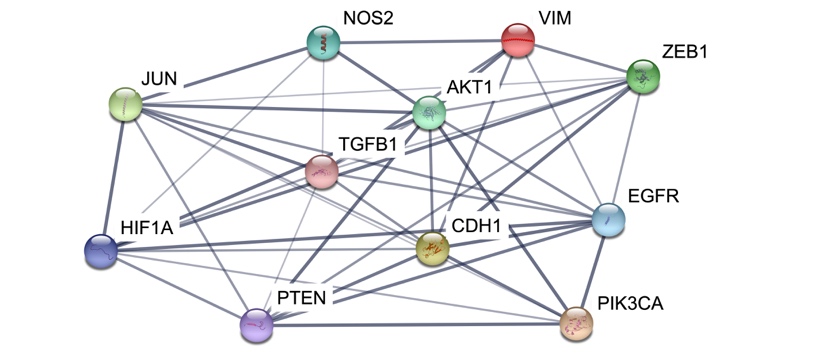


**A**

**Mutual Exclusivity Analysis (PanCancer Atlas Studies), 76639 samples**

**B**


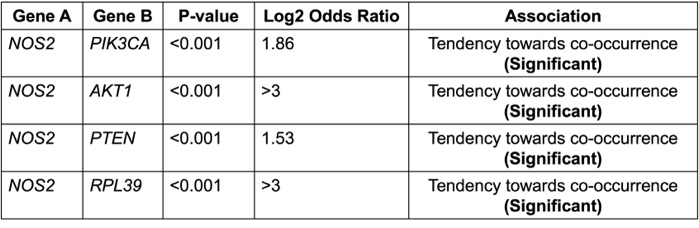


**C**

**Mutual Exclusivity Analysis (Breast Cancer Studies) 8644 samples**


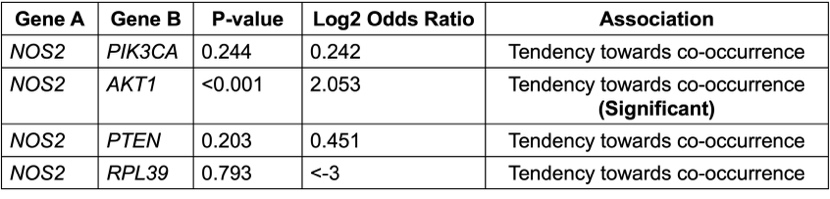


**Supplementary Figure 2. (A)** Network of Protein interactions with iNOS, PI3K, EMT, and hypoxia-related genes generated by STRING analysis at high confidence. Each network node represents one gene. **(B)** Mutual Exclusivity Analysis of cBioPortal Combined Dataset of PanCancer Atlas (n=76639) and **(C)** Breast Cancer Studies (n=8644) comparing genomic alteration co-occurrence/mutual exclusivity of *NOS2* with *PIK3CA, AKT1, PTEN*, and *RPL39* molecular alterations. mRNA expression and protein/phosphoprotein level data profiles were selected in analysis. Statistical analysis using one-sided Fisher’s Exact Test.

**Supplementary Figure** 3


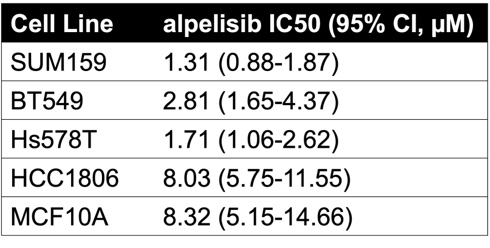

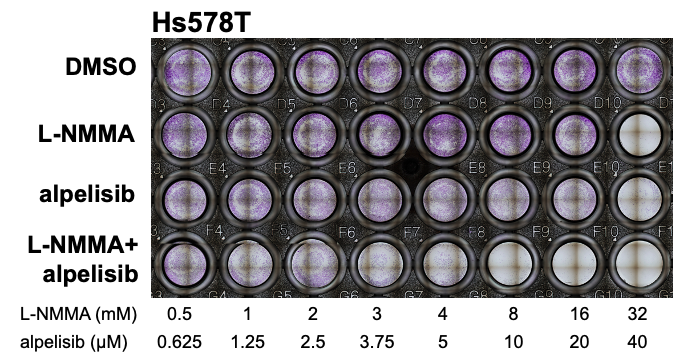

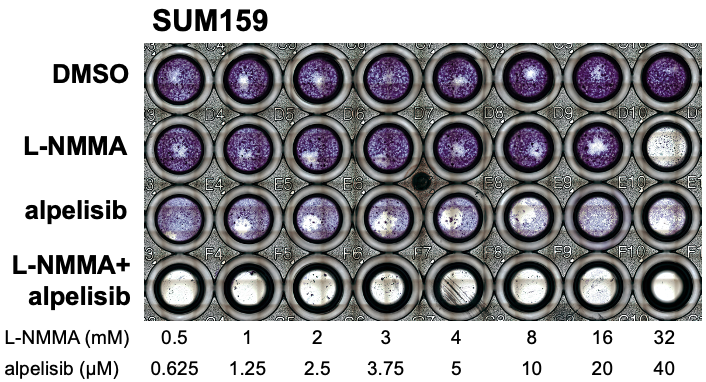

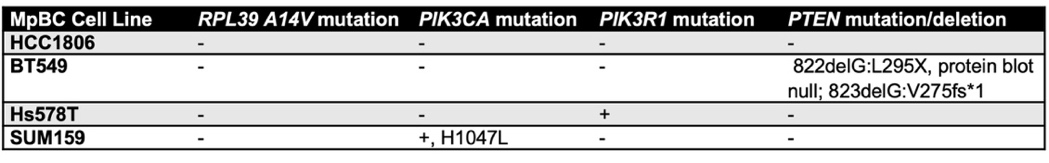


**B**

**C**

**A**


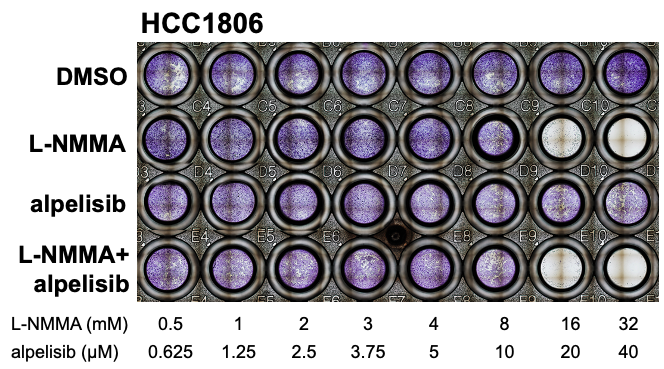

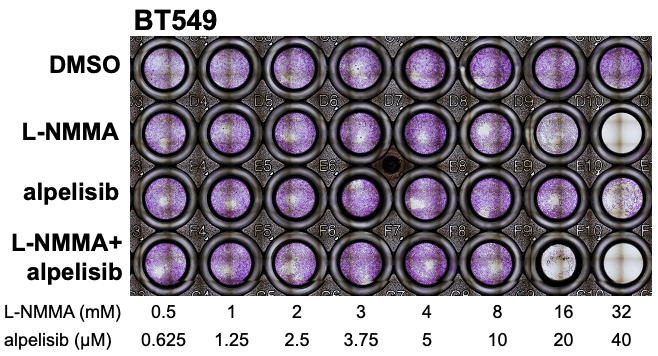


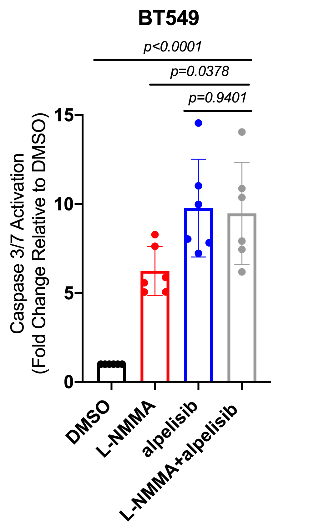

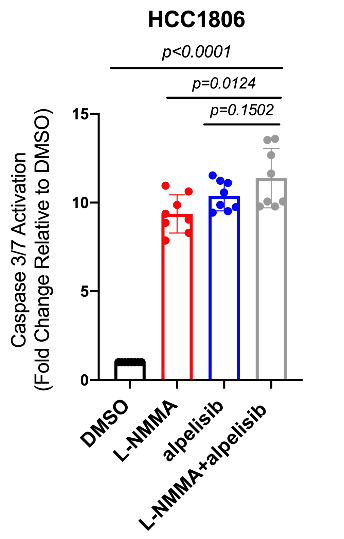

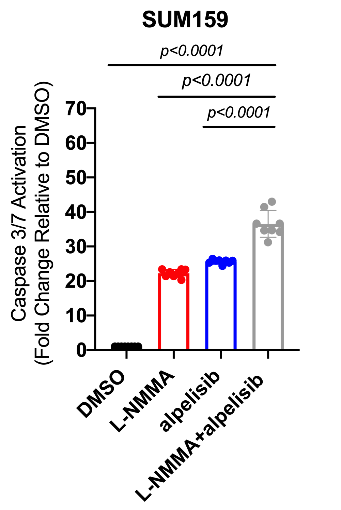

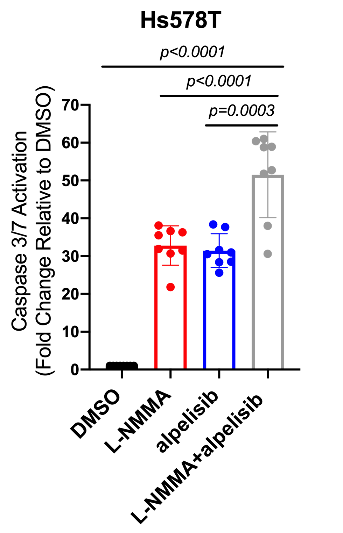


**D**


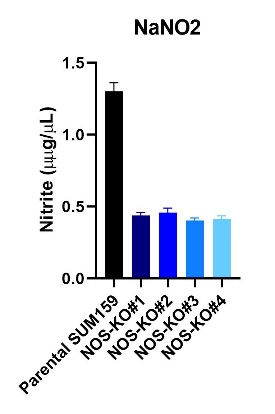

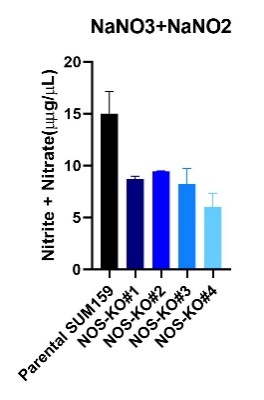


**E**


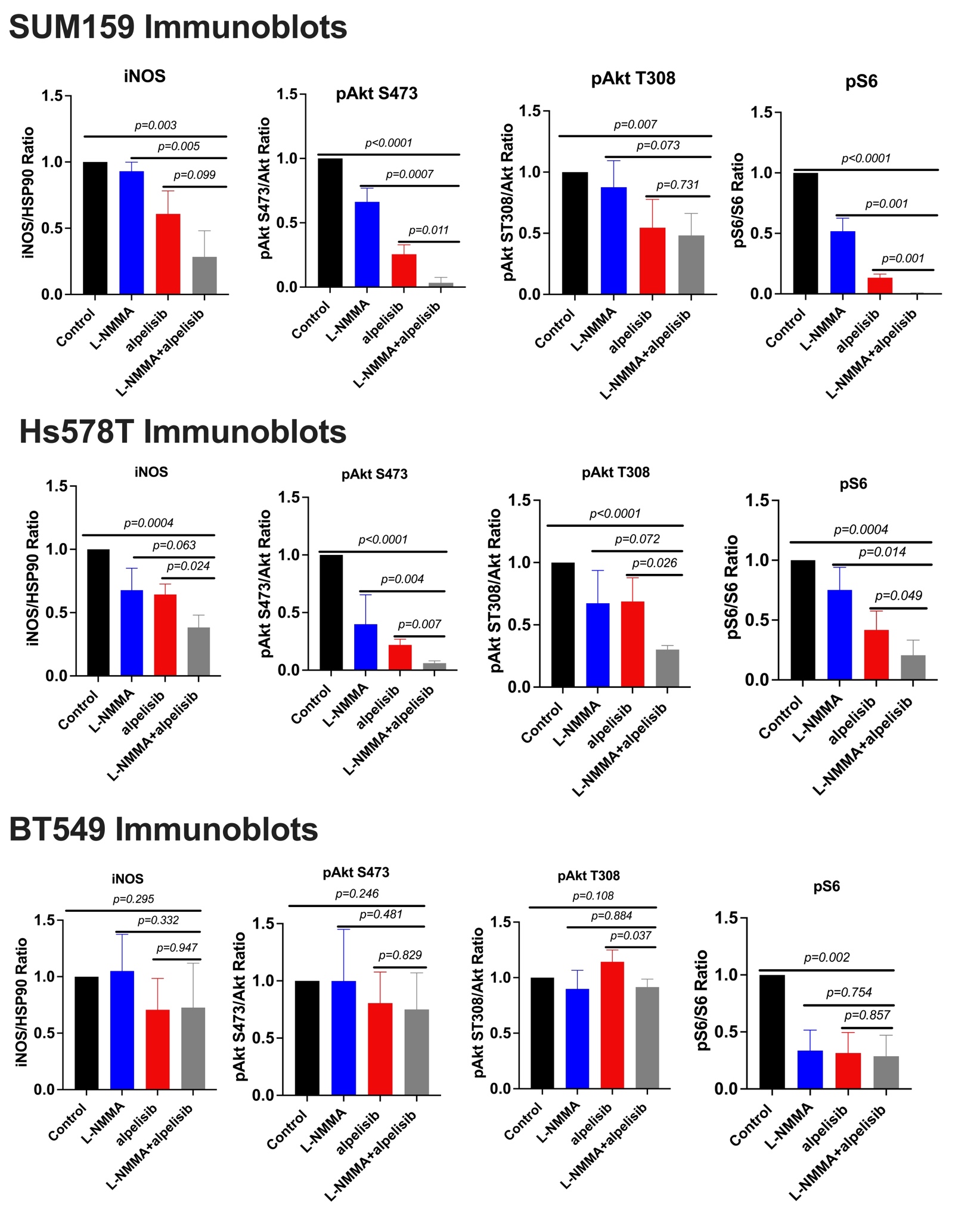

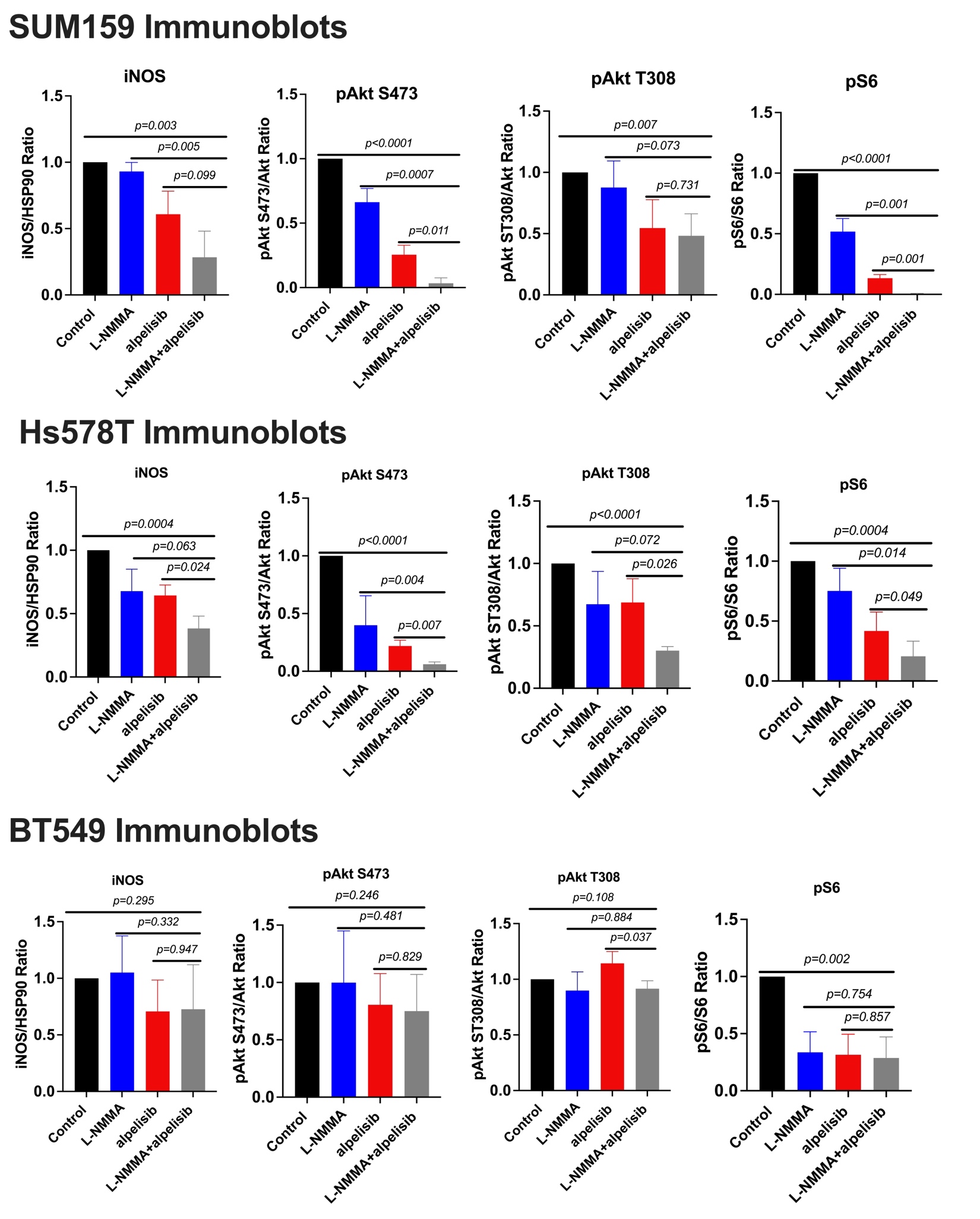

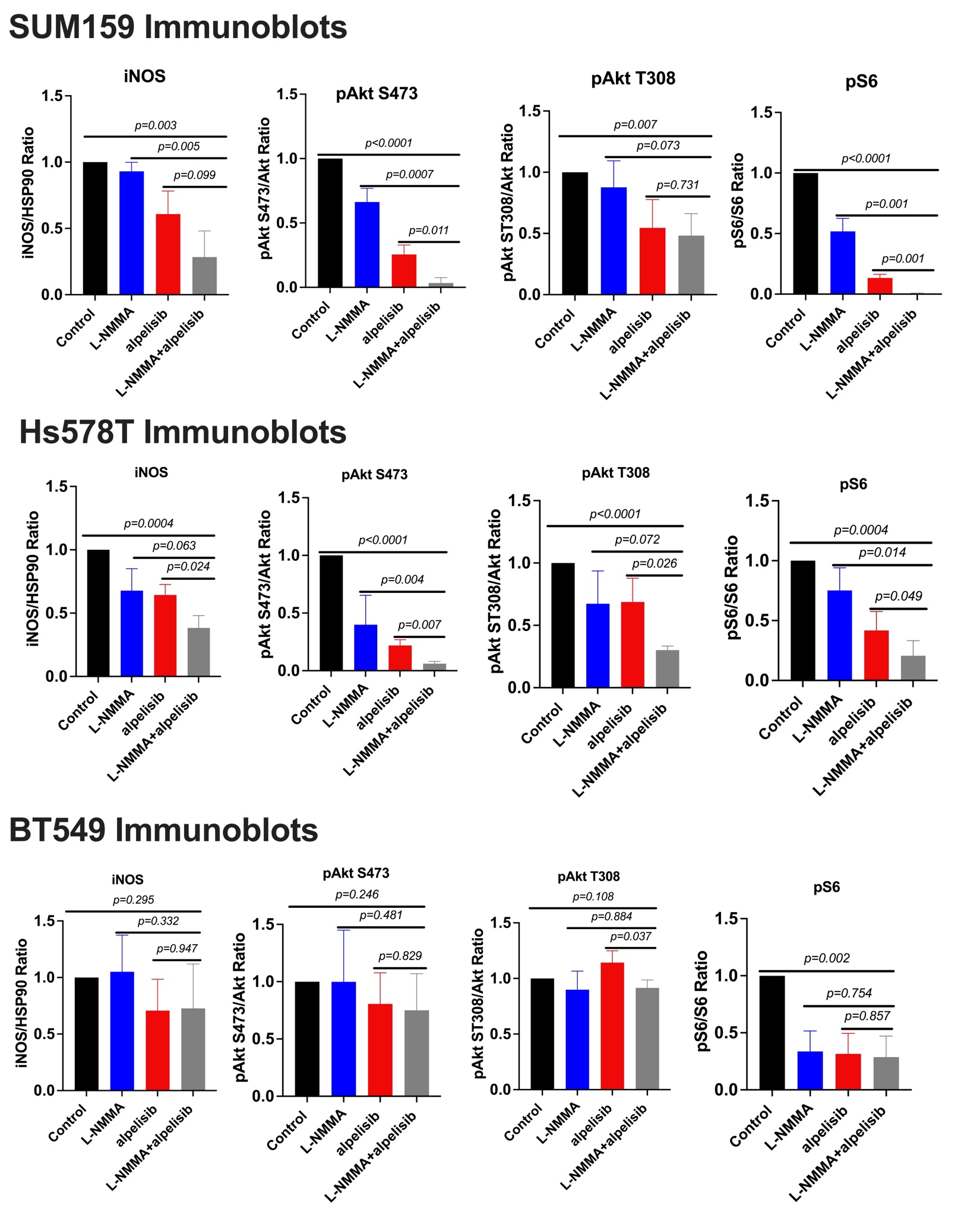


**F**


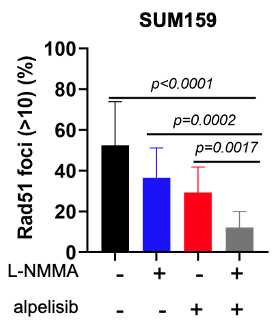

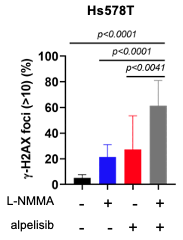

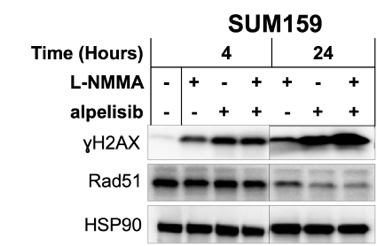

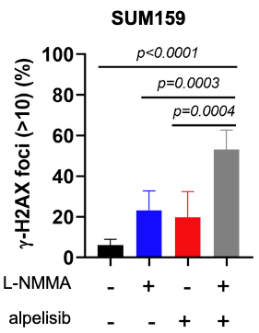

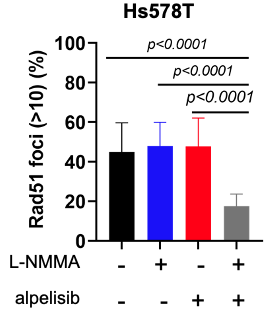

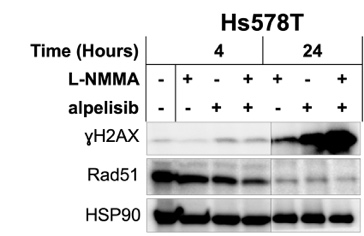

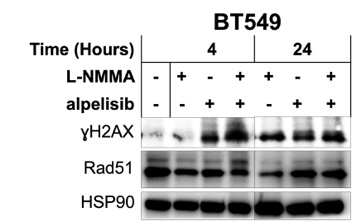


**G**

**H**

**I**

**
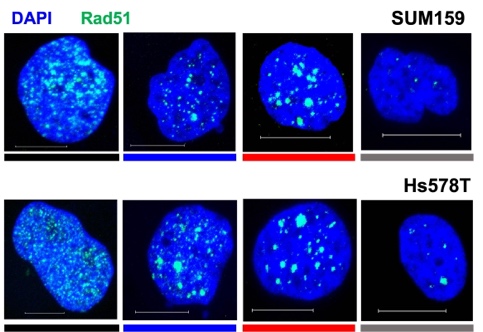
**

**
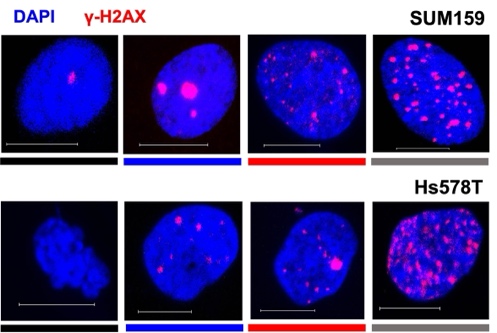
**


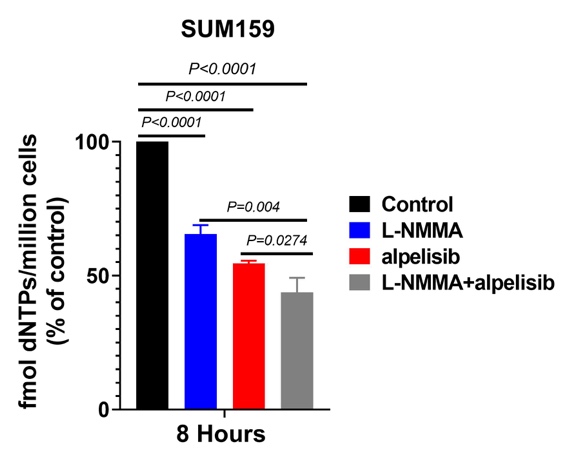

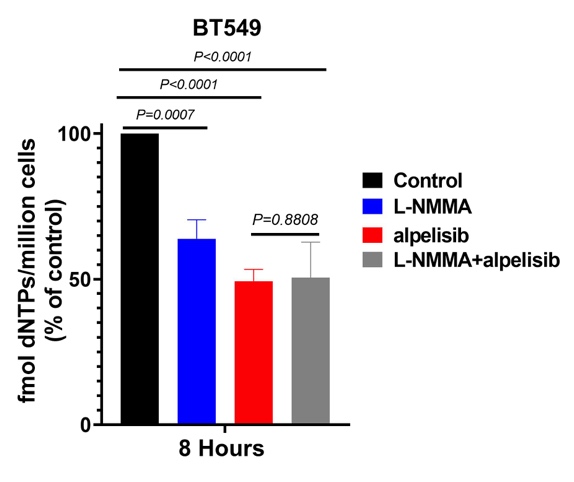


**K**

**J**

**SUM159**

**BT549**

**Supplementary Figure 3. Pan-NOSi L-NMMA enhances PI3Ki induced DNA damage in MpBC cell lines with *PIK3CA/PIK3R1* mutations (A)** *RPL39* and PI3K pathway gene alterations in MpBC cell lines. **(B)** Individual IC_50_ values of alpelisib in MpBC cell lines as determined by Cell Titer Glo Assay and IC_50_ values were determined using PRISM software. **(C)** Crystal violet staining of anchorage-dependent colony formation assay indicates the sensitivity of cells to DMSO, L-NMMA, alpelisib, or alpelisib combined with L-NMMA. Effect of treatments is shown for SUM159, Hs578T, BT549, and HCC1806 cell lines. **(D)** Quantification of caspase 3/7 activation over a 72-hour time course of 4 mM L-NMMA and 5 µM alpelisib. Data displayed are representative of 6-8 biological replicates and fold change of activation is compared to fluorescence values obtained from DMSO treatment arm. In the graphs, DMSO control is indicated in black, L-NMMA in red, alpelisib in blue, and L-NMMA+alpelisib in grey. **(E)** Nitrite/Nitrate values in SUM159 control and NOS2KO cells, as determined by colorimetric nitrite/nitrate assay kit (Sigma). (**F**) Densitometry analysis of immunoblots shown in Figure 3D quantified using ImageLab Software (Biorad). Statistical analysis by Student’s *t* test. Analysis was performed from immunoblots of three independent experiments. (**G-H**) Immunofluorescence analysis of Rad51 foci **(G)** and γH2AX foci **(H)** formation. Images were captured at 100X magnification with confocal microscope. Scale bars represent 15 µM. Statistical analysis by Student’s *t* test. n=3. **(I)** Immunoblotting of γH2AX and Rad51 in SUM159 (*PIK3CA* mutated), Hs578T (*PIK3R1* mutated), BT549 (*PTEN* deleted cell lines treated for 4-24 hours with DMSO control, 4 mM L-NMMA, 5 µM alpelisib, and L-NMMA combined with alpelisib. **(J-K)** dNTP quantification results from competitive PCR of BT549 **(J)** and SUM159 **(K)** cells treated for 8 hours with inhibitors. Statistical analysis by two-sided Student’s *t* test.

**Supplementary Figure** **4**


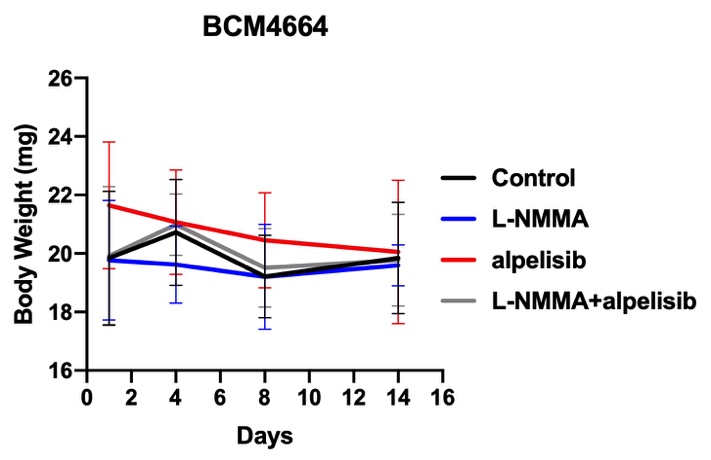

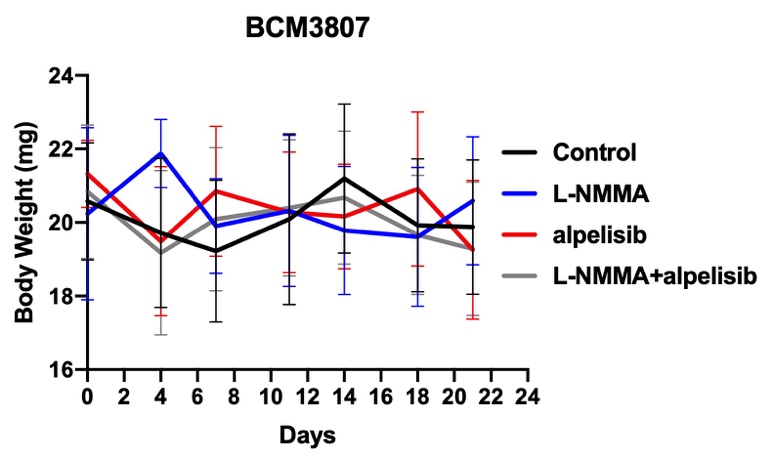


**B**

**A**


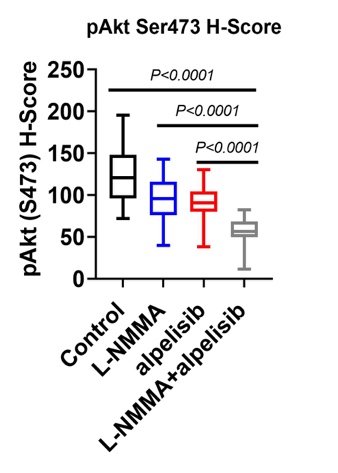

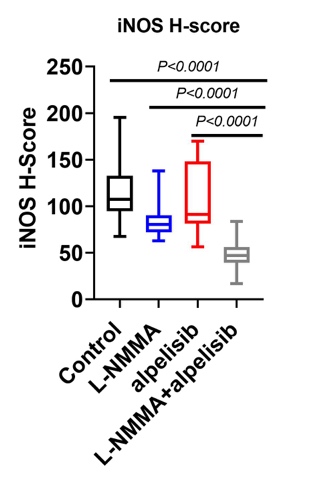

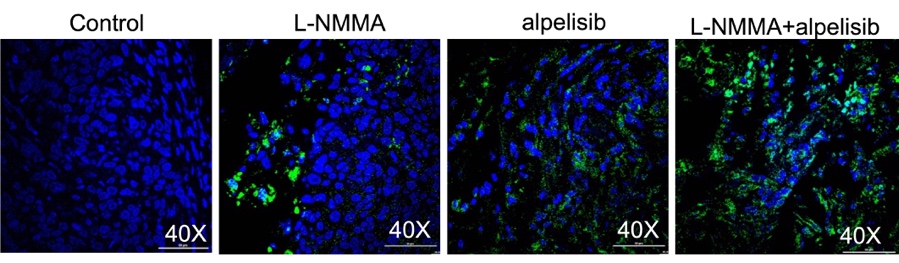


**C**

**D**

**BCM-3807 Tumors**

**E**

**F**


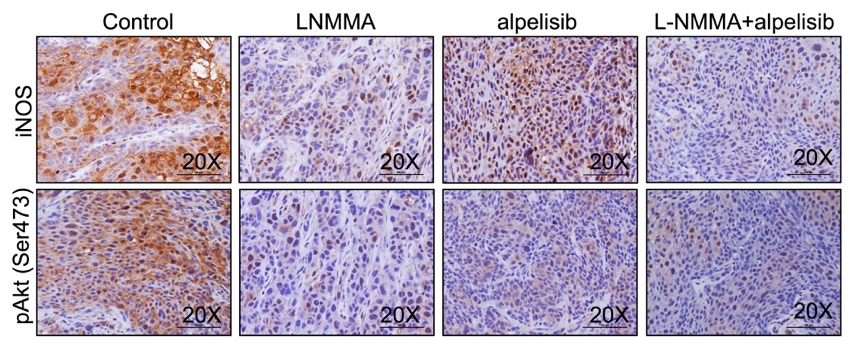


Cleaved Caspase 3 DAPI


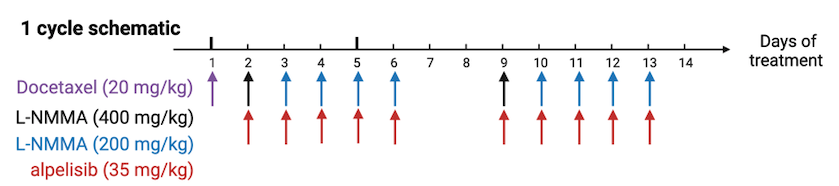


**G**


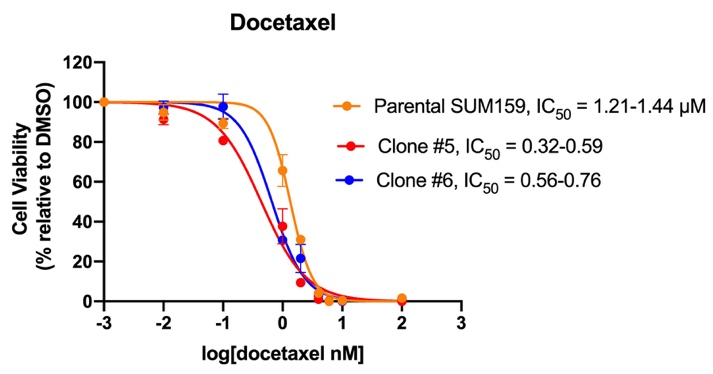


**H**

**Supplementary Figure 4. (A-B)** Body weights of mice implanted with BCM-4664 (**A**) and BCM03807 (**B**) tumors during treatment with inhibitors. **(C)** Representative images of immunohistochemistry (IHC) for iNOS and pAkt in single agent versus combined agent treated MpBC PDX tissues. Scale bars represent 100 µM. **(D)** H-score quantification results of iNOS and **(E)** pAkt (Ser473) IHC staining from tumor tissues. H-scores were evaluated in ten image fields per tissue sample (n=3/treatment arm). Statistical analysis by two-sided Student’s T test. **(F)** Images of immunofluorescence stain of cleaved caspase 3 and DAPI nuclear stain in single-agent versus combined agent treated MpBC PDX tissues. Scale bars represent 100 µM. **(G)** Schematic representation of MpBC PDXs (BCM-3807 [*PIK3CA* mutant] and BCM-4664 [*PIK3CA* WT]) experimental design. PDXs derived from human MpBC were transplanted into cleared mammary fat pad of female NSG mice. When tumors reached 150-200 mm^3^, mice were randomized to receive vehicle control, docetaxel (20 mg/kg on day 1), NOS inhibition therapy (L-NMMA [400 mg/kg oral gavage on day 1, 200 mg/kg oral gavage on days 2-5] + amlodipine [10 mg/kg intraperitoneal injection on days 1-5]), PI3K inhibitor alpelisib (35 mg/kg oral gavage on days 1-5), or the combination of therapies as indicated. Caliper measurements were taken twice a week. **(H)** Cell Glo Titer Cell Viability Assay results of SUM159 control and NOS2KO clones treated with docetaxel at varied concentrations for 72 hours. IC_50_ values were determined by GraphPad Prism software.

**Supplementary Figure** **5**


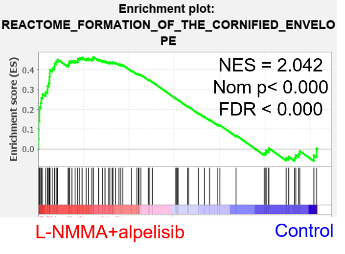

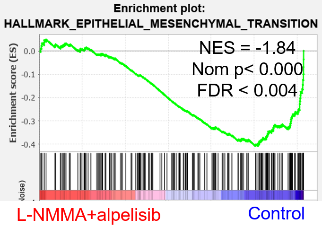

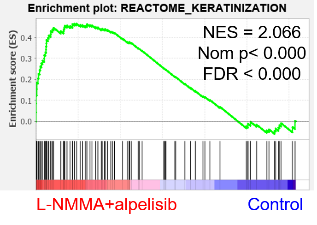


**A**

HALLMARK EPITHELIAL TO MESENCHYMAL TRANSITION

REACTOME FORMATION OF CORNIFIED ENVELOPE

REACTOME KERATINIZATION


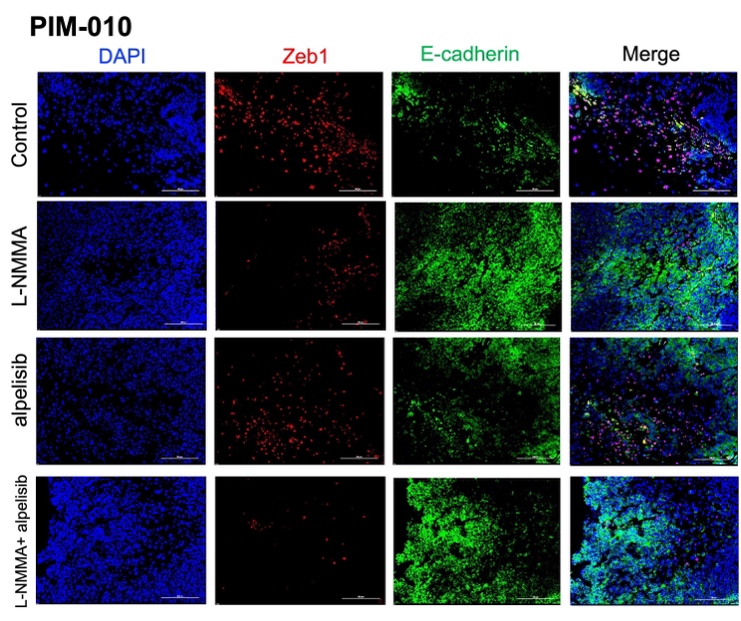


**C**

**B**


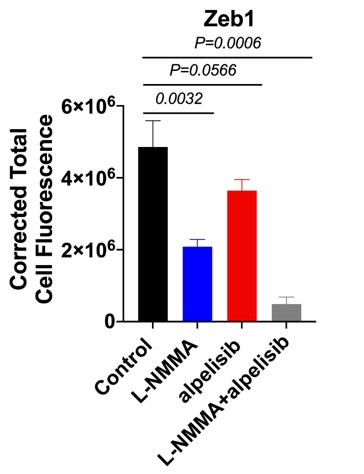

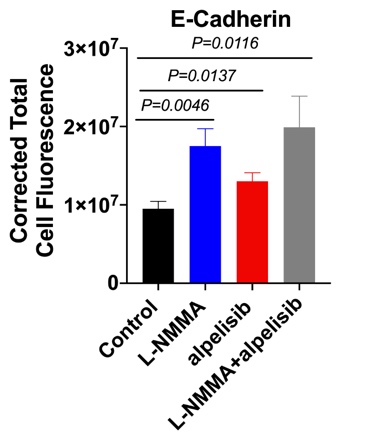


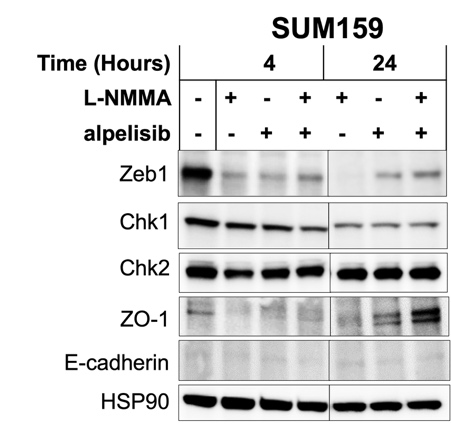

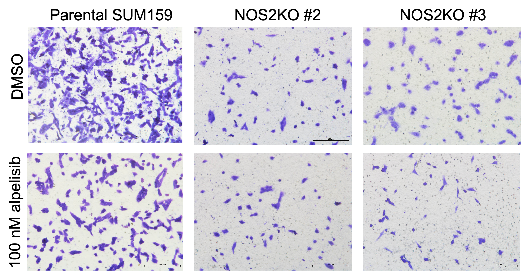

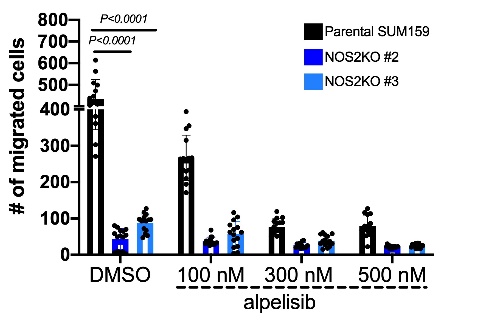


**D**

**F**

**E**


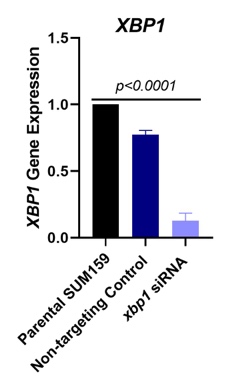

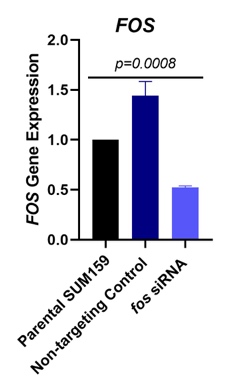

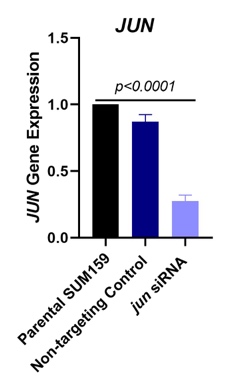

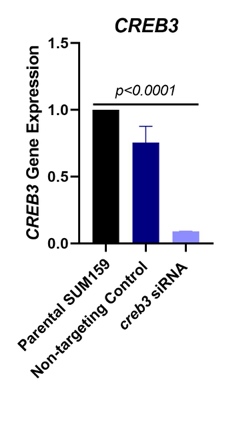


**I**


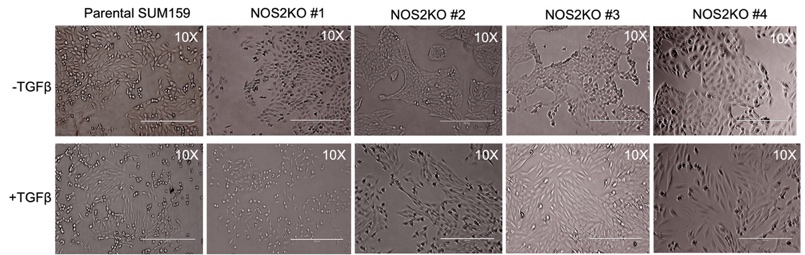


**G**


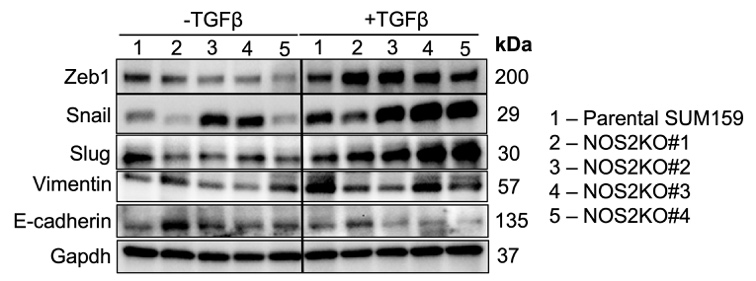


**H**


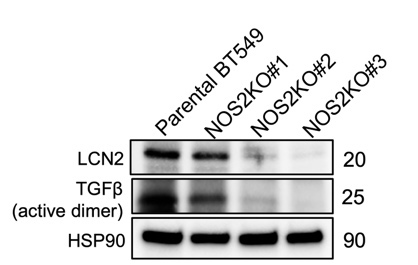

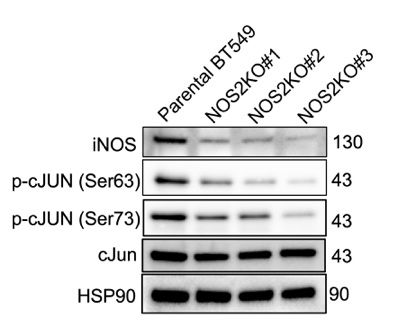

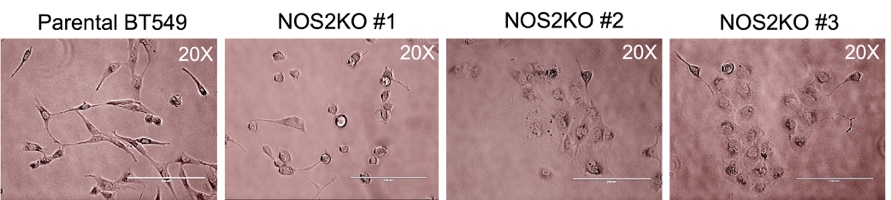


**L**

**J**

**K**

**
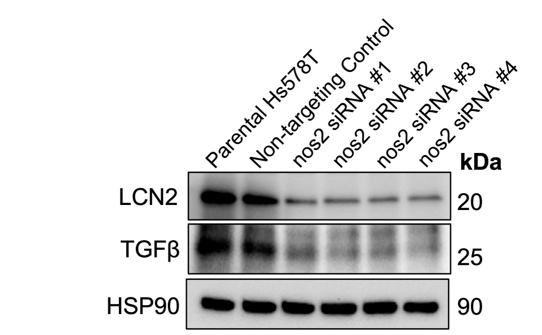

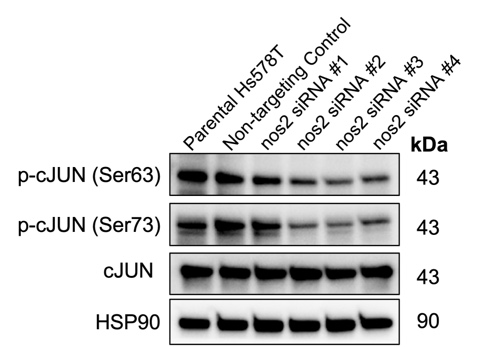

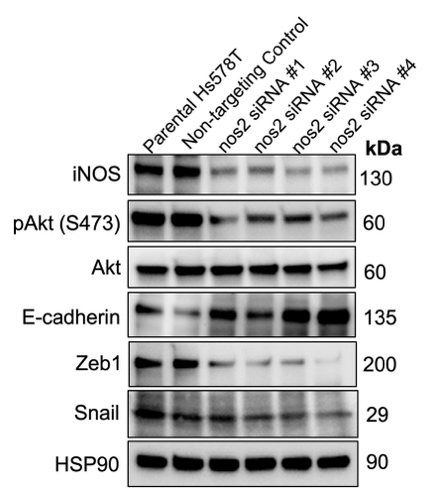
**

**N**

**O**

**M**

**Supplementary Figure 5**. **(A)** GSEA plots of keratinization, formation of the cornified envelope, and EMT pathways. NES, nominal p value, and false discovery rate (FDR) are indicated. **(B)** Representative immunofluorescence images of PIM-010 tumors evaluated for **(C)** E-cadherin and Zeb1 protein expression. Statistical analysis by Student’s *t* test. n=3. Images captured at 10x magnification at scale bars represent 200 µM. **(D)** Immunoblotting of EMT markers in SUM159 (*PIK3CA* mutated) cell lines treated for 4-24 hours with DMSO control, L-NMMA, alpelisib, and L-NMMA combined with alpelisib. **(E)** Migration of Parental SUM159 and NOS2KO cells following treatment with vehicle (DMSO) or different concentrations of alpelisib for 24 hours. Scale bars represent 500 µM. **(F)** Graph indicates the mean of migrated cells per field ± SD of three independent experiments; two-tailed Student *t* test. **(G)** Representative images of cell morphology of Parental SUM159 and NOS2KO clone cells treated with TGFβ and **(H)** corresponding immunoblots of mesenchymal transcription factors and Vimentin from untreated/TGFβ-treated cells. 20x magnification and scale bars represent 200 µM. **(I)** mRNA expression of *XBP1, CREB3, FOS,* and *JUN* from qPCR analysis of Parental SUM159 cells and cells treated with non-targeting control siRNA and gene-specific siRNAs. Statistical analysis by Student’s *t* test. n=3 replicates. (**J and K**) Immunoblots of **(J)** LCN2, TGFβ [active form], **(K)** phospho-cJun (Ser63/Ser73) and HSP90 loading control in Parental BT549 cells and NOS2KO clone cells. **(L)** Morphology of BT549 control and NOS2KO clone cells. 20x magnification and scale bars represent 200 µM. **(M)** Immunoblots of EMT and iNOS associated proteins, (**N**) phospho-cJun (Ser63/Ser73) and HSP90 loading control, and (**O**) LCN2, TGFβ [active form], and HSP90 loading control in Hs578T cells, and Hs578T cells treated with non-targeting control, and siRNAs specific to NOS2 for 96 hours. Bars and error bars represent the mean ± SD of three biological replicates. For all Blots, images shown are representative of n=3 biological replicates.

**Supplementary Figure** **6**


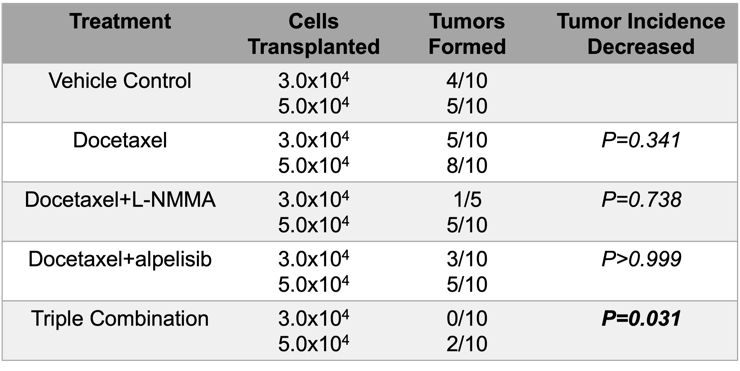


**A**

**B**


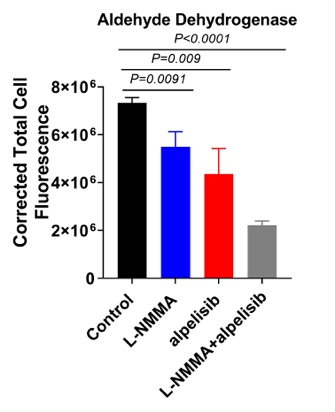

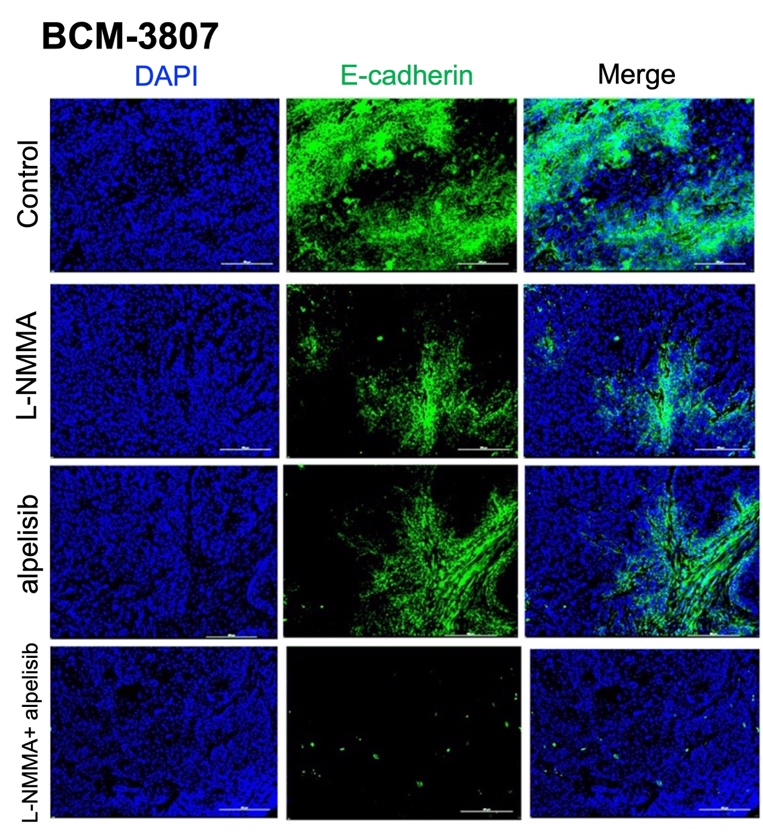


**C**


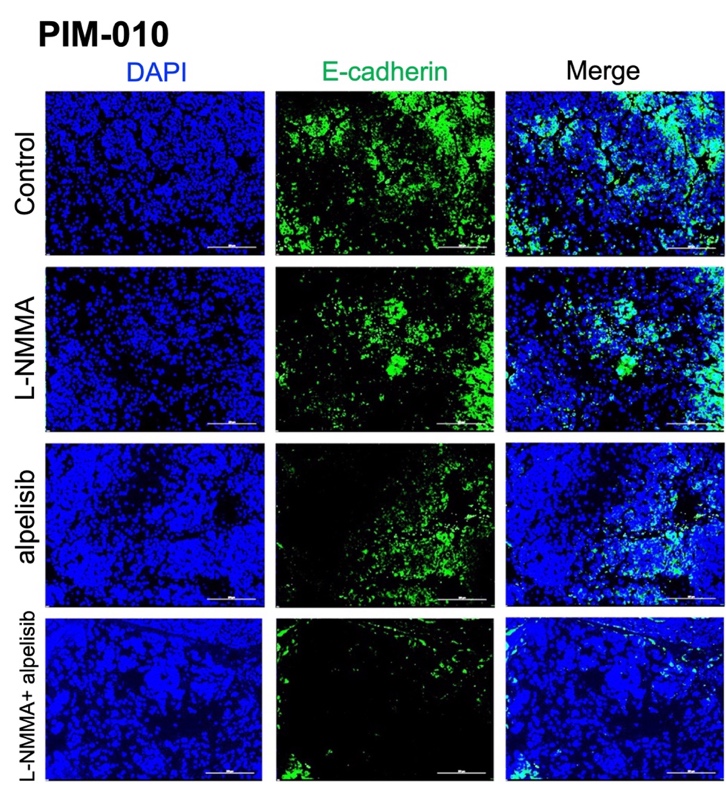


**D**

**E**


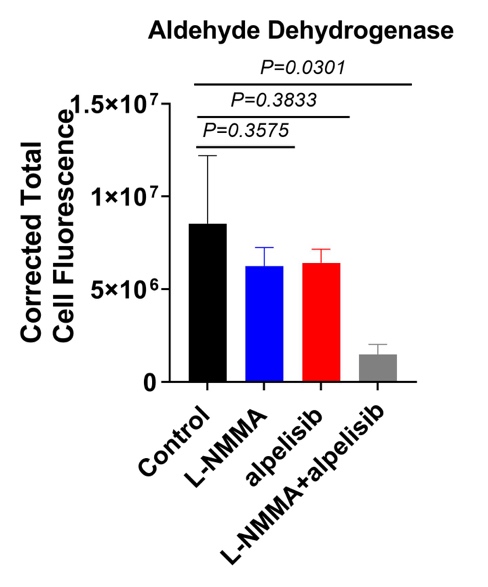


**Supplementary Figure 6. (A)** Limiting Dilution Assay: PDX-3807 tumors from vehicle- and treated mice were dissociated and pooled. A total of 30,000 or 50,000 cells from each group were transplanted into the mammary gland fat-pad of 4- to 6-week-old mice. Tumor incidence was reported at 12 weeks post-transplantation. Data were analyzed by pairwise comparisons with Fischer exact test. **(B-E)** Representative immunofluorescence images **(B)** of BCM-3807 and (**D**) PIM-010 tumors evaluated for ALDH1 protein expression with (**C and E**) corrected total cell fluorescence. Statistical analysis by Student’s *t* test. n=3 mice per treatment arm. Images captured at 10x magnification and scale bars represent 200 µM.

**Supplementary Figure** **7**


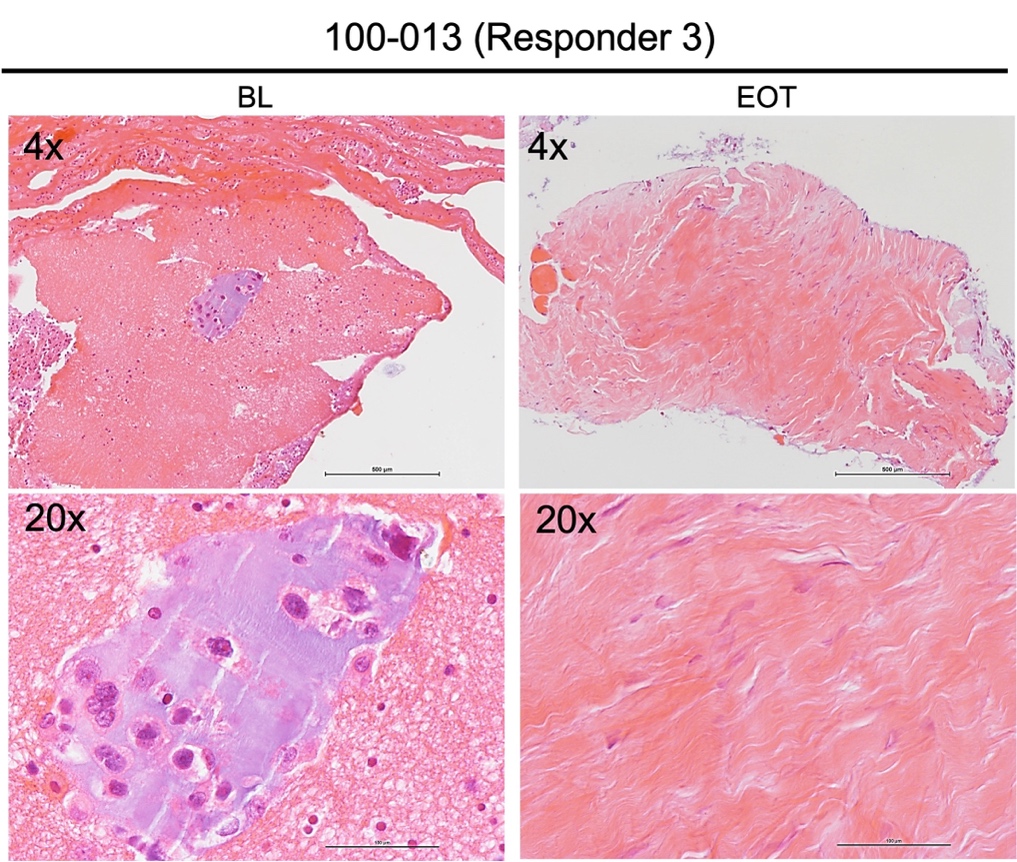

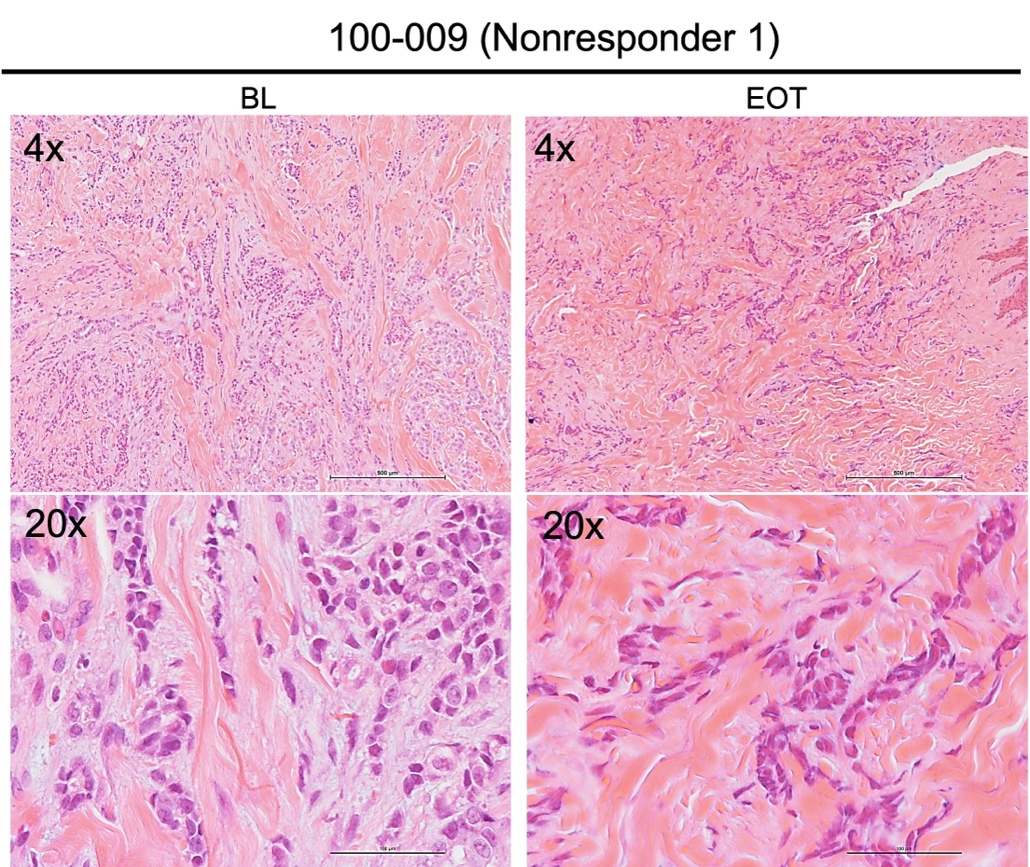


**A**

**B**

**Supplementary Figure 7.** Representative images of Hematoxylin and Eosin staining of baseline tumor from responder 100-013 **(A)** and non-responder 100-009 **(B)** and end-of-treatment tissue. Light microscope images captured at 4x and 20x using a Nikon eclipse 90i microscope with a DS-Fi1 bright field camera (Nikon Instruments Inc). Scale bars for 4x and 20x image are 500 µM and 100 µM, respectively.

**Supplementary Figure** **8**


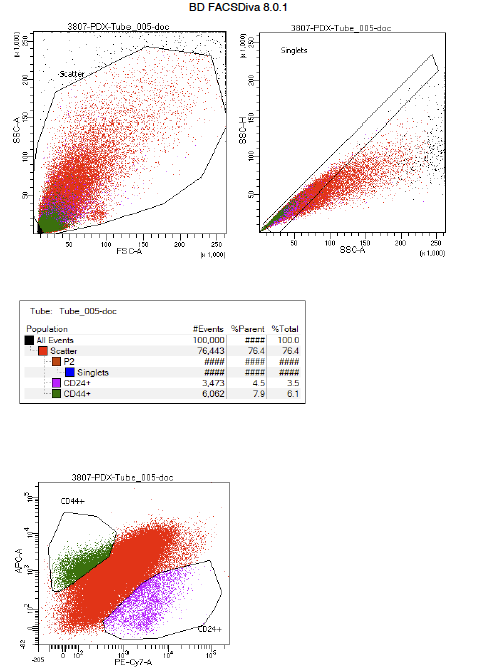
 A


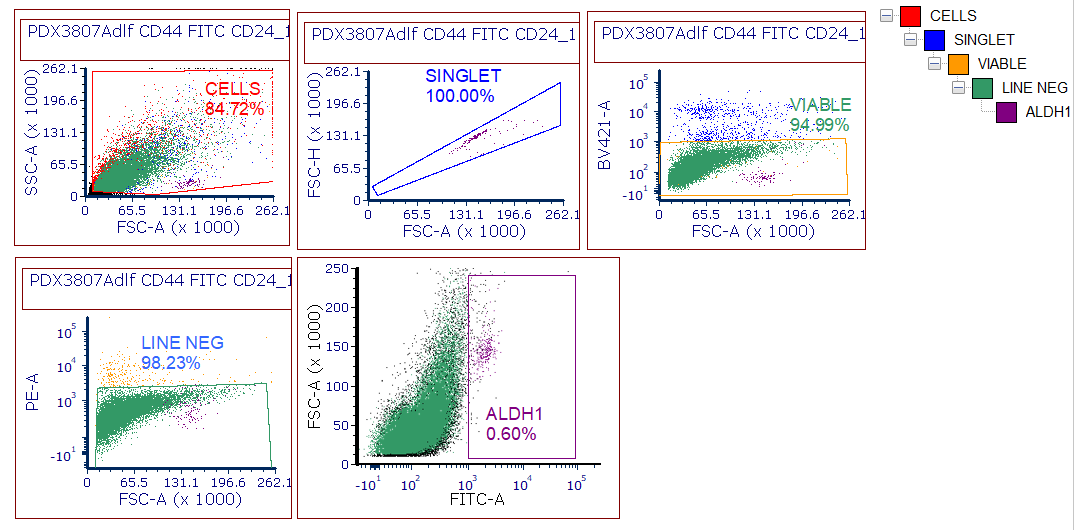


B

**Supplementary Figure 8.** **(A)** Example of gating strategy for flow cytometry analysis of breast cancer stem cells (CD44+/CD24-) derived from metaplastic breast cancer patient derived xenograft tissues used in Figure 6D. **(B)** Example of gating strategy for flow cytometry analysis of breast cancer stem cells (ALDH1+) derived from metaplastic breast cancer patient derived xenograft tissues used in Figure 6G.
